# Psychosocial risk and protective factors for youth problem behavior are associated with food addiction in the Generation Z

**DOI:** 10.3389/fpubh.2024.1414110

**Published:** 2024-05-27

**Authors:** Luisa Mastrobattista, Luis J. Gomez Perez, Luigi Gallimberti, Bruno Genetti, Alessandra Andreotti, Daniele Fassinato, Lucia Monacis, Pasquale Anselmi, Daiana Colledani, Adele Minutillo, Claudia Mortali

**Affiliations:** ^1^National Centre on Addiction and Doping, Italian National Institute of Health, Rome, Italy; ^2^Novella Fronda Foundation, Padua, Italy; ^3^Explora Research and Statistical Analysis, Padua, Italy; ^4^Department of Humanities, University of Foggia, Foggia, Italy; ^5^Department of Philosophy, Sociology, Education and Applied Psychology (FISPPA), University of Padova, Padua, Italy

**Keywords:** food addiction, psychosocial risk and protective factors, behavioral addiction, adolescence, Generation Z

## Abstract

**Objective:**

Food Addiction (FA) and other well-known risk behavior as substance misuse tend to co-occur and may share similar risk and protective factors. The aim of this study was to assess the association between the diagnosis/severity of FA and psychosocial domains typically related to risk behavior syndrome in a large, nationally representative community sample of Generation Z underage Italian students.

**Method:**

The sample consisted of 8,755 students (3,623 from middle schools, 5,132 from high schools). A short version of the Yale Food Addiction Scale 2.0 was administered to evaluate FA. Risk and protective factors related to demographic, personality, behavior, and family variables were examined. Stepwise multivariate logistic and linear regressions were conducted.

**Results:**

The prevalence of FA was 30.8%. Female gender, social anxiety and depression symptoms, social withdrawal risk, Internet gaming disorder, social media addiction, current substance use, social challenge engagement and experienced doxing boosted the chance of FA diagnosis, whereas eating fruit and vegetables, playing competitive sports and an average sleep duration of 7–8 h per night reduced these odds. FA severity was significantly and positively associated with trait impulsiveness, social anxiety and depressive symptoms, risk of social withdrawal, recent substance use, social media, and gaming addiction, doxing suffered and risky social challenges participation. Negative associations between the severity of FA and fruit and vegetable diet habits were found.

**Conclusion:**

Our findings confirm that FA is widespread among Italian adolescents. The associations between the diagnosis and severity of FA and psychosocial risk factors for health, including, addictive and deviant behaviors related to digital misuse, suggest its belonging to the risk behavior constellation. Health promotion schemes based on a multicomponent strategy of intervention should consider the inclusion of FA and its psychosocial correlates.

## 1 Introduction

The increased availability and intake of hyper-palatable foods in the last decades (1) has raised important concerns since it is considered a relevant contributor to the spreading risk of obesity and overweight in both children and adults (2, 3). Indeed, large cohort longitudinal studies have found high daily consumption of ultra-processed foods to be associated with greater boosts overtime on indicators of child and adolescent adiposity (4).

Growing evidence has consistently related this escalation in consumption with the potential addictive role of these foods (5, 6), suggesting that food reward-related ability could promote not only this raising trend but also trigger addiction symptoms in a similar manner to substance use disorders (SUD) (7–9). For instance, one of the most frequently referred antecedents of binge eating episodes is craving for sweets (10) or carbohydrates (11). Craving is a SUD core feature considered a strong predictor of drug use (12). Interestingly, recent neuroimaging studies have revealed common neural underpinnings for craving induced by food and drug cues (13).

The Yale Food Addiction Scale (YFAS) (14) is a self-reported scale developed to assess addiction signs to ultra-processed foods by adapting the Diagnostic and Statistical Manual of Mental Disorders IV (DSM-IV) criteria for substance dependence to “Food Addiction” (FA). Lately, this version has been updated into the YFAS 2.0 (15), which reflects the oncological changes for SUDs underwent in the DSM-5. Systematic reviews of the YFAS studies on adults have reported estimated prevalence of FA ranging from 0 to 25.7% in nonclinical samples and from 6.7% up to 100% in some eating disorder clinical cohorts (16).

This phenomenon also seems to be widespread among children and adolescents. In fact, a recent meta-analysis of the studies assessing FA in this populations reported an average prevalence rate of 12% for community samples and 19% for overweight and obese individuals (17). These rates on adolescence may be particularly troubling since, in this neurodevelopmental phase, the strong influence of motivational substrates is coupled with the immature and not yet fully effective inhibitory control system (18). This could thus determine a heightened vulnerability among youths toward the emergence of psychosocial problems and health risky behaviors as addictive substance use (19) or delinquency (20). Substantial evidence supports the key notion of the adolescent problem behavior model (21) that youth risk behaviors tend to co-occur (22). It is well-known that the juvenile involvement of risk behaviors like aggression is strongly associated with the engagement in other risk behaviors as substance use or crime (23). Moreover, high levels of co-occurrence between risky behaviors in adolescence have been shown to predict negative outcomes in terms of social adjustment in the adulthood (24). Results indicate that problematic food and substance consumption often occur together (25) and symptoms of FA are positively correlated with smoking, alcohol, and cannabis use among adolescents (26). This suggests that underage FA could be part of the constellation or syndrome of risk behavior (27), and therefore, shares similar risk and protective factors. Within this framework, several psychosocial domains are relevant to the prediction of adolescents problematic life-styles (28, 29): among others, sociodemographic, family, personality and behavioral risk factors. Sociodemographic factors as lower socioeconomic status (SES) have been shown to be associated with externalizing problems (withdrawn and aggressive behavior) (30). Family variables as the quality of the father-child relationship have been found to be a predictor of the risk of engagement in multiple teen risky behaviors (31). Key personality features as impulsivity, negative emotionality, avoidant tendencies, and other personality risk factors reflecting psychosocial unconventionality as low academic achievement have been linked to a higher likelihood of involvement in multiple adolescent problem behaviors (32, 33). Regarding the behavioral domain, recently, the adolescent risk behaviors related to the overuse of the Information and communication technologies (ICTs) are being a matter of growing concern because of the particularly increasing usage, especially among adolescent of Generation Z (born from 1997 through 2012), since the beginning of the COVID-19 pandemic (34, 35). In this generation, also known as “digital natives” because of their early immersion in socio-digital technologies (36), ICTs misuse and online deviant behaviors have been found to be associated with offline teen risky behaviors as drug use or sexual risk behaviors (37–40).

Alongside with behavioral risk factor, the social-psychological framework has considered the involvement in conventional and healthy behavior as serving to attenuate the impact and effects of risk factors (41). Indeed, health behaviors as balanced diet or regular sleep habits and conventional activities as the participation in sports have been shown to be protective against multiple risk-taking behavior in Generation Z adolescents (42, 43). Therefore, this risk and protective factors approach provides an explanatory schema for studying changes in multiple risk behavior among youth and vulnerable groups (27) that could be useful to develop effective prevention and treatment strategies. Furthermore, this idea underlies the application of this psychosocial approach to the study of new variants of adolescent risk behaviors among digital natives, such as problematic cell phone use (44), social networking site usage (37) or excessive videogaming (45).

Hence, given the association of FA with other well-known risk behaviors such as substance abuse among youth and the commonalities from a clinical and neurobiological standpoint, it could be helpful to test whether and to what extent, these problematic behaviors share similar risk and protective factors. Thus, the aim of the present research was to assess the association between the diagnosis/severity of FA and psychosocial domains widely evinced as related to behavioral health risk factors (29) in a large and nationally representative community sample of Italian underage students. An additional purpose was to determine the prevalence of this phenomenon (i.e., FA) among adolescents from Italy. Our hypothesis is that typical psychosocial risk and protective factors of the risk behavior syndrome will fulfill the same function in predicting the prevalence and severity of FA.

## 2 Materials and methods

### 2.1 Participants

A national survey called “Generation Z” and aimed to assess behavioral addiction and other mental health problems among 11–17-year-old Italian students was conducted by the Italian National Institute of Health during the 2022 academic year at middle and high schools allocated throughout the Italian territory.

A 3-stage probability-proportional-to-size (PPS) sampling procedure was adopted to ensure the best possible representativeness of the population under study (i.e., 11–17-year-old Italian students). For stratification, the municipalities were considered as the first stage unit, the schools as the second stage unit and the school classes as the third stage unit. At the first stage, the municipalities (45 for middle schools and 55 for high schools) were selected according to a stratification level by geographic macro-area (North West, North East, Central Italy, South and Islands) and by municipality size (200,000 inhabitants and more, between 100,000 and 199,999 inhabitants and less than 100,000 inhabitants). Within each first-stage stratum, at the second stage middle schools (71) were selected with a probability proportional to the number of students enrolled; high schools (82) were stratified by type of school (high schools, vocational schools, technical institutes and art institutes) and selected with a probability proportional to the number of students enrolled. At the third stage, classes were selected with probability proportional to the number of students enrolled in the class.

To warrant a sample size of at least 4,000 questionnaires for each age range (i.e., 11–13 and 14–17-year-old) and hypothesizing a participation rate of between 15 and 20%, 676 middle and high schools across the national territory were invited to take part on a voluntary basis. The participation in the study was 22.7% on the part of middle schools (21.1% in North West, 23.9% in North East, 26.2% in Central Italy, 21.4% in the South, and 20.0% in the Islands) and 22.6% on the part of high schools (23.3% in North West, 26.8% in North East, 18.5% in Central Italy, 21.1% in the South, and 22.6% in the Islands). A total of 10,181 questionnaires were collected (4,140 and 6,041 in middle and high schools respectively), of which 1,426 (14.0%) were discarded due to respondents outside the target age (under 11 years or over 17 years), incomplete responses, and questionnaires filled out twice due to technical connection problems.

The final sample was therefore composed of 8,755 students (mean age = 14.03 years, SD = 1.98), 3,623 (41.4%) of which were attending middle school (mean age = 11.99 years, SD = 0.81) and 5,132 (58.6%) were high school pupils (mean age = 15.48 years, SD = 1.08).

Females (4,187, 47.8%) and males (4,291, 49.0%) were equally distributed throughout the sample. A small proportion of students preferred not to report their gender (277, 3.2%). Most of the participants (7,535, 86.1%) reported being of Italian nationality.

Written informed consent was required from all enrolled students and their parents. The collection and processing of the data was carried out in accordance with the national privacy regulations to ensure anonymity. The study was approved by the National Ethical Committee of the Italian National Institute of Health (prot. PRE BIO CE 0010655 of 22/03/2022). All procedures were in accordance with the 1964 Helsinki declaration and its later amendments.

### 2.2 Instruments

All students were presented with a questionnaire which assessed FA and the main domains of risk factors associated with the most common health risk behaviors in adolescents (29). The survey was administered electronically, in the classroom during class time, and in the presence of a trained experimenter who was instructed to assist students if necessary.

#### 2.2.1 Food addiction assessment: the short form of Yale food addiction scale 2.0 (S-YFAS 2.0)

The S-YFAS 2.0^1^ is short form of the Italian version of the YFAS 2.0 (46) validated on large sample of middle and high school Italian students, proving to be an efficient and sensitive measurement of FA. The scale consists of 24 items, scored on an 8-point Likert scale (i.e., from 0 = “*never*” to 7 = “*every day*”), accounting for 11 symptoms of addiction-like eating behavior plus the perceived level of distress derived from them, reported over 1-year period. No sum score is calculated from the single items but each criteria (i.e., symptom and distress criteria) is considered present when at least one of the two questions concerning it reaches its own specific threshold [established by Receiver Operator Characteristic (ROC) curves; (15)]. Like the long version, the S-YFAS 2.0 allows two scoring modalities: a symptom score reflecting the number of symptom criteria that are met and ranging from 0 to 11; and a dichotomous diagnosis which is defined as the endorsement of two or more symptom criteria in addition to the criterion of clinically significant distress.

#### 2.2.2 Sociodemographic risk factors

Demographic variables comprised age, gender, nationality, and region of residence, which was employed to compute the Subnational Human Development Index [SHDI; (47)] according to the data retrieved from the Subnational HDI Database.^2^ The SHDI, used here as socioeconomic status proxy, is an average measure at the subnational level of the education, health, and standard of living indexes whose score ranges from 0 to 1. Higher values indicate greater human development (48). To facilitate data interpretation, the SHDI value was categorized based on the percentile distribution provided by the aforementioned database as follows: ≤ 85th percentile/90th percentile/ 95th percentile.

#### 2.2.3 Family risk factors

The quality of the familiar relationship was assessed by means of the following question with dichotomous response: *How easy is it for you to talk to your mother and/or father about things that really worry you?* (“Easy”; “Difficult or Do not have or see them”). We focused on the assessment of the parent-adolescent communication easiness because it has been associated with high parent–child relationship satisfaction (49), which is deemed to fulfill the role of protective factors from problem behaviors in the Jessor et al.’s theoretical framework (27).

#### 2.2.4 Personality risk factors

As a measure of trait impulsiveness, the Italian version of the Barratt Impulsiveness Scale (BIS-15) (50), was administered. This 15-item self-report scale is an abbreviated version of the Italian BIS–11 (51). This brief variant has not been specifically validated in the Italian adolescent population, although the 30-item version from which it is derived has been adapted to this population (52). As recommended (53), we considered the BIS-15 score as continuous with higher scores indicating higher impulsivity.

The negative emotionality and avoidance factors were assessed throughout the following questionnaires:

- Severity Measure for Social Anxiety Disorder (Social Phobia)— Child Age 11–17 (SAD-D; 54): A 10-item measure evaluating the DSM-5 criteria for social anxiety disorder (54) during the past 7 days and providing a 5-point severity level (i.e., 0 = None/1 = Mild/2 = Moderate/ 3 = Severe/ 4 = Very severe).
- Severity Measure for Depression, Child Age 11 to 17 (PHQ-9 modified for Adolescents [PHQ-A], Adapted) (55): A 9-item measure (score range: 0–27) assessing the severity of clinically significant depressive symptoms during the past week as follows: 0–4 = None, 5–9 = Mild, 10–14 = Moderate, 15–19 = Moderately severe, and 20–27 = Severe.

Although these two emerging APA measures (i.e., the SAD-D and the PHQ-A) have been proposed as useful tools for research and clinical evaluation (54), and some evidence of validity has emerged in adolescent samples (56, 57), they have not been specifically validated in Italian youths.

- Hikikomori Risk Inventory-15 [HRI-15; (58)]: This is a short version of the HRI-24 (59), measuring the typical feelings and behaviors related to social withdrawal on adolescents. A total score (range: 15–75) representing the Hikikomori risk score can be computed and an empirical cut-off score of 37 has been defined for identifying at-risk individuals.

For the analytical and clinical purposes of simplifying as much as possible the interpretation and classification of risk factors (60), the above variables were dichotomized as follows: Social Anxiety Disorder (i.e., SAD-D: None vs. Mild/Moderate/Severe/Very Severe) and Depression Disorder (i.e., PHQ-A: None vs. Mild/Moderate/Moderately severe /Severe). Moreover, a participant with a score of 37 or more in the HRI-15 was considered as at-risk of social withdrawal (58).

Psychosocial unconventionality [i.e., Non-compliance to conventional behavior standards; (58)] was captured in relation to the school institution by assessing the last year academic performance throughout the following question: *How was your academic performance last year?* The following three response options were available: Failed or lower than the class average, on average or higher than the class average, I do not remember.

#### 2.2.5 Behavioral risk factors

Factors associated with teen unhealthy lifestyles (28), such as substance use and other addictions or forms of deviant behavior associated with ICTs use, were considered within the behavioral risk factor domain. Likewise, measures related to more conventional activities, as potential protective factors of risk-taking behaviors (61), were included.

Substance consumption was ascertained by asking participants whether they had consumed alcohol, tobacco, or energy drinks in the last month.

Addictive behaviors related to digital technology were investigated through the following self-reported scales:

- Italian version of Bergen Social Media Addiction Scale [BSMAS; (62)]: A 6-item scale (score range: 6–30) assessing core addiction symptoms related to past year social media use. Recent research suggests a total score of 24 as the optimal clinical cut-off score (63). Therefore, this score was considered as discriminant of Social Media Addiction (SMA) in our study.
- Internet Gaming Disorder scale–short-form [IGDS9-SF; (64)]: A 9-item scale (score range: 9–45) corresponding to the nine core criteria of DSM-5 for Internet Gaming Disorder (IGD) (54) assessed over a 12-month period. In this study, the empirical cut-off point of 21 defined by Monacis et al. (64) was used to establish this diagnosis in our sample.

The main forms of ICTs related deviant behavior contemplated in this survey were:

- Doxing [i.e., internet dissemination without consent of other’s personal and sensitive data; (62)] was checked by asking participants the following queries: *Have you ever shared photos, images, personal data of someone without their consent to make fun of them?* (Yes/No) and *Has anyone ever shared photos, images, or personal data without your consent to make fun of you (excluding your family members)?* (Yes/No).
- Online Self-Harm Challenge engagement [i.e., risk-taking practices mediated by digital sociability; (63)] was investigated through an *ad hoc* dichotomous question about respondents lifetime participation in this kind of challenges (i.e., *Have you ever participated in dangerous online social challenges like “the Skullbreaker challenge” or similar?* Yes/No).

The assessment of conventional lifestyle was based on the following question:

- Dietary habits: *How many times a week do you usually eat fruit or vegetables?* (Never /Not every week/Weekly).
- Sports habits: *Do you play competitive sports?* (Yes/No).
- Volunteering: *Do you often go to parish/ volunteering/ scouting groups?* (Yes/No)
- Sleep habits:
  ■ Sleep duration: *How many hours did you sleep on average at night during the last month?* (6 h or less/7–8 h/9–10 h/More than 10 h).
  ■ Sleep latency: *During the last month, how long did it usually take you to fall asleep each night?* (Less than 15 min/15–45 min/More than 45 min).

### 2.3 Data analysis

The FA diagnosis, the S-YFAS 2.0 individual symptoms prevalence, and the mean symptom score were calculated for the total sample.

The primary outcomes of the present study were (1) the FA diagnosis and (2) the symptom score as defined above. The latter dependent variable was selected for being considered as a sensitive indicator of FA severity in non-clinical adolescent populations (65).

Regarding the first primary outcome, a preliminary bivariate analysis was conducted between the FA diagnosis variable and each exposure of the above-described domains. Specifically, concerning the symptom score, the Kolmogorov–Smirnov normality test was performed (for each exposure factors), and the Skewness and Kurtosis indices were verified (|Skewness|, |Kurtosis| > 1). The differences among groups were analyzed via nonparametric tests, namely Mann–Whitney test (2 groups) and Kruskal-Wallis test (3 groups or more). Then, a multivariate logistic regression was used to analyze the association between the outcome variable (i.e., FA diagnosis) and the independent variables which were found to be associated with it in the bivariate model. All these variables were entered as covariates one at a time using a forward stepwise approach. Variables were retained in the model if the *p*-value of the regression coefficient was less than 0.05. Subsequently, all variables were entered as covariates using a backward stepwise approach and removed from the model if the *p* value of the regression coefficient was greater than 0.10. The model with the best goodness of fit (i.e., Cox and Snell pseudo R2) between the two was selected. Odds Ratio (OR) was calculated as an effect size (ES) index of the association between every exposure and the outcome. For the ORs larger than 1, values of 1.22, 1.86, and 3.00 were considered small, medium, and large effect sizes; for the ORs lower than 1, values 0.82, 0.54, and 0.33 were pondered small, medium, and large effect sizes (66).

Thereafter, for examining the psychosocial factors associated with the severity of FA, two multivariate linear regressions, one with the total sample and the other with the subsample of participants with FA diagnosis, were applied with the symptom score as dependent variable and all the variables covering the psychosocial domain mentioned before as covariates. The same back and forward stepwise procedure as described for the multivariate logistic regression model was adopted, except for the model selection method which was based on the higher coefficient of determination (R^2^). Cohen’s *f* ^2^ was calculated for each independent variable in each model as a local ES measure. In line with Cohen’s (67) guidelines, *f* ^2^ ≥ 0.02, *f* ^2^ ≥ 0.15, and *f* ^2^ ≥ 0.35 were considered small, medium, and large effect sizes, respectively.

The statistical analyses were performed using IBM SPSS Statistics 20.

## 3 Results

### 3.1 Descriptive statistics

Prevalence rates of each FA symptoms, of the FA diagnosis, the total sample mean symptom score and the results of the bivariate analysis are depicted in Supplementary Table 1. It is worth noting that the prevalence of FA was 30.8%, with a mean symptom score of 1.5 (SD = 2.4) for the total sample and of 4.3 (SD = 2.6) for the FA diagnosed subsample. Concerning the individual symptoms of FA, “Great deal of time spent” was the most frequent (16.9%). In relation to sociodemographic domain, female gender (40.0% vs. 21.3% of male gender) was more often associated with the FA diagnosis. Regarding family domain, participants who reported difficulties in talking with parents (40.4% vs. 22.3% of those referring parent’s easy talking) more frequently met the FA diagnostic criteria. Within the personality domain, individuals at risk of social withdrawal (76.9%) were much more classified as food addicts than those who were not at risk (29.9%), also reporting high mean symptom scores (mean = 4.5, SD = 3.5). In the behavioral domain, the covariate most associated with increased prevalence of FA was SMA, with a FA diagnosis percentage more than double among participants with SMA (74.6%) compared to those not affected (29.5%). Furthermore, the mean symptom score was more than three times higher in the former (mean = 4.7, SD = 3.7) than in the latter (mean = 1.4, SD = 2.3).

### 3.2 Associations between FA diagnosis and psychosocial risk factors

**Figure 1:**
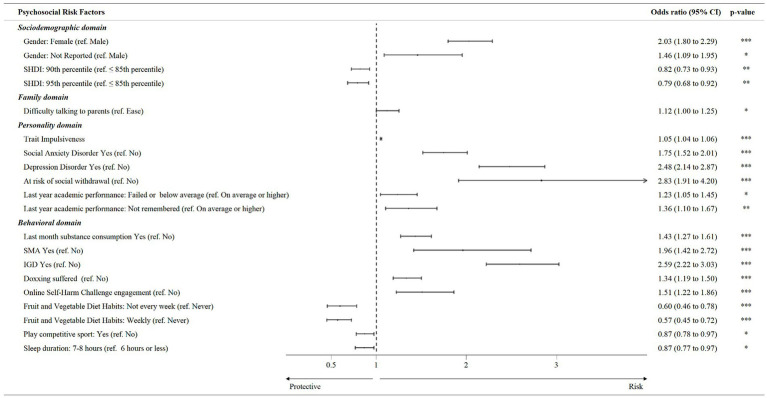
Multivariate logistic regression analysis of associations between Food Addiction diagnosis and psychosocial risk factors.

Figure 1 shows the results of the multivariate logistic regression model. As regard to sociodemographic factors, female gender (OR = 2.03, 95% CI [1.80, 2.29]) and preference of not reporting gender (OR = 1.46, 95% CI [1.09, 1.95]) raised the odds of FA diagnosis, whereas a 95^th^ SHDI percentile (OR 0.79, 95% CI [0.68, 0.92]) and a 90^th^ SHDI percentile (OR = 0.82, 95% CI [0.73, 0.93]) lowered this likelihood. ESs were medium for female gender, small to medium for the preference of not reporting gender, and small for SHDI categories. As to familiar relationship quality, child’s difficult talking with parents (OR = 1.12, 95% CI [1.00, 1.25]) increased the probability of meeting the diagnostic criteria for FA, although the ES for this significant association was small. Respecting to personality features, the presence of social anxiety (OR 1.75, 95% CI [1.52, 2.01]) and depression symptomatology (OR = 2.48, 95% CI [2.14, 2.87]), the risk of social withdrawal (OR = 2.83, 95% CI [1.91, 4.20]), a higher score in a trait impulsiveness indicator (OR = 1.05, 95% CI [1.04, 1.06]) and a last year academic performance below the average (OR = 1.23, 95% CI [1.05, 1.45]) or not remembered (OR = 1.36, 95% CI [1.10, 1.67]) incremented the odds of FA. The ESs of all these associations were small except for social anxiety, depression, and social withdrawal, which were respectively, small to medium, and medium to large. In reference to the behavioral domain, suffering from IGD (OR = 2.59, 95% CI [2.22, 3.03]) and from SMA (OR = 1.96, 95% CI [1.42, 2.72]), last month substance consuming (OR = 1.43, 95% CI [1.27, 1.61]), online self-harm challenge engagement (OR = 1.51, 95% CI [1.22, 1.86]), and being victim of doxing (OR = 1.34, 95% CI [1.19, 1.50]) boost the chance of FA. Instead, eating fruit and vegetables weekly (OR = 0.57, 95% CI [0.45, 0.72]) or even not every week (OR = 0.60, 95% CI [0.46, 0.78]), playing competitive sports (OR = 0.87, 95% CI [0.78, 0.97]), and a monthly average sleep duration of 7–8 h per night (OR = 0.87, 95% CI [0.77, 0.97]) reduced the odds of being classified as addicted to food. The ESs were small for the variables of doxing, competitive sports playing and sleep duration, small to medium for last month substance use, online self-harm challenge engagement and for the frequency of eating fruit/vegetables, and medium to large for the IGD and SMA exposures.

### 3.3 Associations between S-YFAS 2.0 symptom score and psychosocial risk factors in the total sample

**Figure 2:**
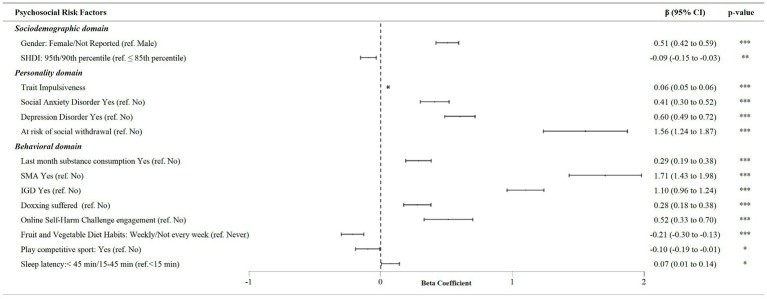
Multivariate linear regression analysis of associations between S-YFAS 2.0 symptom score and psychosocial risk factors in the total sample.

The stepwise multivariate linear regression analysis revealed that S-YFAS 2.0 symptom count was significantly and positively associated with female and the preference of not reporting gender (*β* = 0.51, 95% CI [0.42, 0.59]), social anxiety (*β* = 0.41, 95% CI [0.30, 0.52]) and depression disorder symptoms (*β* = 0.60, 95% CI [0.49, 0.72]), risk of social withdrawal (*β* = 1.56, 95% CI [1.24, 1.87]), trait impulsiveness index higher scores (*β* = 0.06, 95% CI = 0.05–0.06), last month substance use (*β* = 0.29, 95% CI [0.19, 0.38]), presence of SMA (*β* = 1.71, 95% CI [1.43, 1.98]) and IGD (*β* = 1.10, 95% CI [0.96, 1.24]), doxing suffered (*β* = 0.28, 95% CI [0.18, 0.38]), participation in an online self-harm challenge (*β* = 0.52, 95% CI [0.33, 0.70]), and an elevated night sleep latency in the last month (*β* = 0.07, 95% CI [0.01, 0.14]; Figure 2). Negative and significant associations were found, instead, between FA symptom score and high SHDI level (*β* = −0.09, 95% CI [−0.15, −0.52]), weekly or quasi-weekly fruit and vegetables intake (*β* = −0.21, 95% CI [−0.30, −0.13]), and the practice of competitive sports (*β* = −0.10, 95% CI [−0.19, −0.01]; Figure 2). However, the ESs for these associations were small (*f* ^2^ ≥ 0.02).

### 3.4 Associations between S-YFAS 2.0 symptom score and psychosocial risk factors in the FA diagnosed subsample

**Figure 3:**
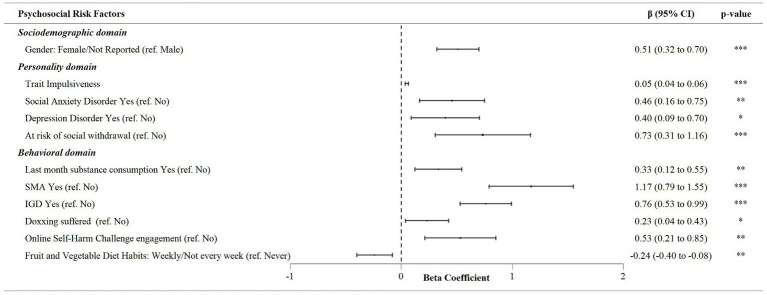
Multivariate linear regression analysis of associations between S-YFAS 2.0 symptom score and psychosocial risk factors in the subsample with Food Addiction diagnosis.

When the psychosocial factors associated with the count of FA symptoms in the subsample of participants with a FA diagnosis were considered, the stepwise multivariate linear regression (Figure 3) found gender (i.e., female and the preference of not reporting gender versus male gender; *β* = 0.51, 95% CI [0.32, 0.70]), the trait impulsiveness index score (*β* = 0.05, 95% CI [0.04, 0.06]), social anxiety (*β* = 0.46, 95% CI [0.16, 0.75]) and depressive symptoms (*β* = 0.40, 95% CI [0.09, 0.70]), risk of social withdrawal (*β* = 0.73, 95% CI [0.31, 1.16]), substance use in the previous month (*β* = 0.33, 95% CI [0.12, 0.55]), SMA (*β* = 1.17, 95% CI [0.79, 1.55]) and IGD diagnosis (*β* = 0.76, 95% CI [0.53, 0.99]), being a doxing victim (*β* = 0.23, 95% CI [0.04, 0.43]) and self-harm social challenge participation (*β* = 0.53, 95% CI [0.21, 0.85]) as significant and positive associated factors of FA disorder severity. Only the fruit and vegetable diet habits (i.e., weekly, or quasi-weekly versus never; *β* = −0.24, 95% CI [−0.40, −0.08]) were observed to be negatively and significantly associated with the severity of this addiction (i.e., higher symptom count on S-YFAS 2.0). It is important to note that the ESs calculated for these associations were small (*f* ^2^ ≥ 0.02).

## 4 Discussion

The study results provide critical information on the prevalence of FA in the largest and nationally representative sample, to our knowledge, of underage students from a European country. Similarly, this research assessed the association between the diagnosis and severity of FA and psychosocial domains related to the risk behavior syndrome (28), considering among these, those particularly raised among Generation Z adolescents during the COVID-19 pandemic period such as addictive and deviant behaviors associated with ICTs (34, 35).

The prevalence of FA in our sample was particularly high (30.8%). As far as we know, this prevalence ranks among the highest reported on children and adolescent samples (17). In a recent meta-analysis (17) analyzing 22 studies and involving a total of 6,996 adolescents, the estimated prevalence of FA was 15% (95% CI [11, 19]) for all samples, 12% (95% CI [8, 17]) for community samples, and 19% (95% CI [14, 26]) for overweight/obese samples. In Italy, in particular, prevalence studies have recently been carried out only in small clinical samples of adolescents with eating disorders yielding rates between 49.4% (68) and 51.9% (69). Our results suggest prevalence rates higher than those above reported on average for the clinical population, which might be due to differences in diagnostic threshold criteria. The studies analyzed in this systematic review used the YFAS, which has a more restrictive diagnostic boundary than the YFAS 2.0 used in our study (70). However, in our study, 17% of food-addicted individuals exhibit moderate (meeting at least 4 criteria) or severe symptoms (meeting at least 6 criteria). Furthermore, among those diagnosed, the symptom count average was higher than 3, which is the diagnostic threshold adopted by the YFAS.

A possible explanation for the high prevalence rate of FA found in our study could be related to the reported increase in habitual consumption of ultra-processed food and physical inactivity among Italian adolescents during the SARS-CoV-2-pandemic period (71–73). Since extant literature has consistently conceived FA as an addiction to this kind of hyper-palatable foods (74), it would not be surprising to suppose that the aforementioned increase in consumption has led to higher rates of addiction. Interestingly, the rise in the intake of these groceries has been strongly linked to boosted boredom and emotional overeating (75). Boredom has had a reinforcing effect of the perceived emotional distress during the pandemic outbreak (76) and emotional overeating, a firmly-related feature of FA (77), could have been an important strategy to cope with it.

Be that as it may, this FA rates, which are specific for population in the adolescent life stage, should raise concerns because of their deleterious impact on youth quality of life dimensions such as physical, emotional, social, and school functioning (78). Furthermore, the early onset of these risk behaviors during adolescence could lead to more severe mental health pathology during adulthood, as it has been demonstrated in the case of substance abuse (79, 80), behavioral addictions (81) and eating disorders (82).

Various associations with FA diagnosis/severity emerged in the psychosocial domains typically related with risk-taking behavior among youth people. Regarding sociodemographic variables, our analysis revealed that higher SHDI values were associated with lower prevalence rates and symptom count of FA. SHDI is a statistical composite index that considers SES indicators as the income *per capita* level. These data seem to be in line with a large body of literature indicating a connection during adolescence between low SES and health risk behaviors as poorer diets, less physical activity, and greater cigarette smoking (83). Interestingly, among adolescents, socio-economic position has been shown to be inversely associated with two closely phenomena for FA (84) as obesity (85) and a high frequency of consumption of fast food (86).

In our study, other demographic variables, such as gender, have shown to be related with FA. Specifically, female gender showed a significantly higher probability of FA diagnosis and greater severity compared to males, drawing attention to previous research [i.e., (87, 88)] highlighting gender differences in addiction tendencies. This result is also supported by Leary et al. (89), who found an association between symptom count and female gender.

Several biological, psychological, and social mechanisms could account for the higher probability of FA in females. For example, it has been suggested that the effects of pubertal ovarian hormones may lead to increased binge eating (90), which has been found to be strongly correlated with FA (91). Moreover, women have shown more brain reactivity to external food-related stimuli in craving-related cerebral regions (92). From a psychosocial perspective, gender differences in FA rates may be connected to gender differences in mental health and body perception (93). Indeed, adolescent girls tend to report higher levels of body dissatisfaction and depression than their male counterparts (94). The negative evaluation of one’s own body and depression symptoms are known risk factors for FA (95, 96).

Concerning the family domain, communication difficulties with parents were significantly associated with FA diagnosis, foregrounding the potential impact of family relationships not only on conventional risk behaviors among youth (31) but also on adolescent eating behaviors as reported in other recent studies (97).

Personality traits such as social anxiety, depressive symptoms, and a tendency for social withdrawal were strongly linked to a higher probability of meeting FA diagnostic criteria. These findings are consistent with the reported associations between anxious-depressive symptoms and FA diagnosis/severity among adolescents (98). This, in turn, appears to be coherent with the evidence showing the interconnectedness of this negative affect symptomatology and multiple domains of teen risk-taking behavior including drug and alcohol use, worse perception of health, computer overuse, academic failure, or overweight (99, 100). Regarding the association between FA and social withdrawal, as far as we are aware, this is first research in reporting it. There is no literature confirming this, but it could reflect a trend toward emotional dysregulation and avoidance as reported in previous studies on FA (101) and other risky behavior samples (32).

In the behavioral domain, several factors related to youth problematic lifestyle increase the risk of diagnosis and are associated with greater FA symptomatology, including IGD, SMA, recent substance use, involvement in risky online challenges, and being a victim of doxing. Indeed, in the literature, FA seems to be associated with substance use (26) or other behavioral addictions (102). This could partly have to do with the online exposure to risky behavior content and its relationship with drug use, excessive alcohol use, disordered eating, self-harm, violence to others, and dangerous pranks, as demonstrated in young adult samples (103). Intriguingly, a significant association between exposure to social media content and disordered eating was only found for female gender, which in our data was a strong predictor of FA (103). In this sense, systematic reviews of the evidence from the field of eating disorders and health psychology hint that mass media are a key source of information and reinforcement regarding the relevance of the thin beauty ideal, and the way to achieve it, determining therefore a media-mediated pressure to be slim that may be a risk factor for body dissatisfaction, weight concerns, and disordered eating behaviors in adolescent girls (104).

On the other hand, our research found that habits such as regular consumption of fruits and vegetables, participation in competitive sports, and adequate sleep duration seemed to have a protective effect against FA similar to that reported in large samples of Generation Z adolescents in the case of other more conventional risk behaviors such as substance use, risky sexual behavior or deviant behavior (42, 43). This suggests that health promotion approaches focusing on these healthy habits could be a potentially effective primary prevention strategy for this type of problematic eating.

In the subgroup diagnosed with FA, similar trends persisted, reinforcing the impact of gender, impulsive tendencies, mental health problems, addictive behaviors, and negative online experiences on the severity of FA. However, the consumption of fruits and vegetables showed an inverse relationship, albeit with a small ES, suggesting a potential pathway to manage the severity of FA symptoms.

The present study has some limitations that should be considered when interpreting our results. First, the cross-sectional nature of the research limits establishing causal relationships between FA and identified correlates. Longitudinal studies could provide a more comprehensive understanding of the temporal relationship between risk factors and the development of FA over time. Second, our study primarily relies on self-reported data, which might be subjected to recall or social desirability bias. Participants might under or over report their eating habits or behavioral tendencies due to perceived societal norms. However, to mitigate this potential bias, we guaranteed that respondents would remain anonymous. Another study limitation lies in the absence of some measures or factors that could contribute to the FA phenomenon such as anthropometric parameters (i.e., BMI, height, weight), genetic and cultural influences, comorbidities, or environmental influences like marketing strategies for hyper-palatable foods. Finally, some of the instruments measuring personality risk factors (i.e., the BIS-15, the SAD-D and the PHQ-A) have not been specifically validated for the target population of our study (i.e., Italian adolescents), although they have shown evidence of validity in adolescent samples from other countries (56, 57) or have been drawn from longer measures actually validated in Italian adolescents (52). This lack of specific validation has been suggested as a possible restraint to cross-cultural comparisons (105).

In any case, these results collectively underline the multifaceted nature of FA, highlighting the importance of considering various psychosocial factors to understand its prevalence and severity among adolescents. Addressing these complex interactions between behavior, environment, and individual characteristics is crucial in developing targeted interventions aimed at preventing and managing FA in this vulnerable demographic group.

In conclusion, as expected, FA is related with conventional psychosocial risk and protective factors of the risk behavior syndrome. Particularly, the association between FA and indicators of deviant and/or dangerous behaviors (i.e., current substance consumption, IGD, SMA, social challenge, doxing) might suggest that FA is part of a cluster of problematic and risky behaviors for health (drug use, academic failure, crime, etc.) in adolescence that prevention interventions should consider.

## Data availability statement

The datasets presented in this article are not readily available because of the sensitive nature of the information included, in compliance with national legislation. Requests to access the datasets should be directed to CM, claudia.mortali@iss.it.

## Ethics statement

The studies involving humans were approved by National Ethical Committee of the Italian National Institute of Health (prot. PRE BIO CE 0010655 of 22/03/2022). The studies were conducted in accordance with the local legislation and institutional requirements. Written informed consent for participation in this study was provided by the participants’ legal guardians/next of kin.

## Author contributions

LM: Conceptualization, Methodology, Writing – original draft, Writing – review & editing, Supervision, Validation. LP: Conceptualization, Methodology, Supervision, Writing – original draft, Writing – review & editing. LG: Supervision, Writing – original draft, Writing – review & editing. BG: Data curation, Formal analysis, Supervision, Writing – original draft, Writing – review & editing. AA: Data curation, Formal analysis, Writing – original draft, Writing – review & editing. DF: Data curation, Formal analysis, Writing – original draft, Writing – review & editing. LM: Supervision, Writing – original draft, Writing – review & editing. PA: Supervision, Writing – original draft, Writing – review & editing. DC: Supervision, Writing – original draft, Writing – review & editing. AM: Conceptualization, Supervision, Writing – original draft, Writing – review & editing. CM: Funding acquisition, Project administration, Supervision, Writing – original draft, Writing – review & editing.

## Footnotes

^1^Anselmi P, Colledani D, Monacis L, Gómez Pérez LJ, Genetti B, Andreotti A, et al. Development and Validation of a Short Form of the Yale Food Addiction Scale 2.0. Manuscript Submitted publication. 2023.

^2^https://globaldatalab.org/shdi/, version v7.0.
