# Supplementary material for: Psychosocial risk and protective factors for youth problem behavior are associated with food addiction in the Generation Z
Source: Front Public Health. 2024 May 27;12:1414110. doi: 10.3389/fpubh.2024.1414110 (PMC11163117; doi:10.3389/fpubh.2024.1414110)
Supplement: Supplementary file 1 [file Table_1.DOCX]

***Supplementary Material***

**Psychosocial risk and protective factors for youth problem behavior are associated with Food Addiction in the Generation Z**

**Luisa Mastrobattista, Luis J. Gomez Perez*, Luigi Gallimberti, Bruno Genetti, Alessandra Andreotti, Daniele Fassinato, Lucia Monacis, Pasquale Anselmi, Daiana Colledani, Adele Minutillo, Claudia Mortali**

*** Correspondence:** Luis J. Gomez Perez: luis.gomez@novellafronda.it

1. **Supplementary Tables**

**Suppl. Table 1:** S-YFAS 2.0 (Yale Food Addiction Scale 2.0-short form); prevalence of individual symptoms, prevalence of food addiction diagnosis and mean symptom score in the entire sample and stratified by psychosocial risk factors.

|  | Food addiction diagnosis^a^ | Consumed more than planned ^a^ | Unable to cut down or stop^a^ | Great deal of time spent^a^ | Important activities given up^a^ | Use despite physical/emotional consequences | Tolerance^a^ | Withdrawal^a^ | Use despite interpersonal/social consequences^a^ | Failure in role obligations^a^ | Use in physically hazardous situations^a^ | Craving^a^ | Clinically significant impairment/ distress^a^ | Mean symptom score (SD)^b^ |
| --- | --- | --- | --- | --- | --- | --- | --- | --- | --- | --- | --- | --- | --- | --- |
| Total (n = 8755) | 30.8% | 13.1% | 14.0% | 16.9% | 14.5% | 15.6% | 13.1% | 13.0% | 16.5% | 10.1% | 10.3% | 10.6% | 13.5% | 1.5 (2.4) |
| ***Sociodemographic domain*** |  |  |  |  |  |  |  |  |  |  |  |  |  |  |
| Gender |  |  |  |  |  |  |  |  |  |  |  |  |  |  |
| Male  (n = 4291) | 21.3%** | 10.0%** | 7.2%** | 12.2%** | 7.5% | 10.1%** | 8.6%** | 7.0%** | 7.4%** | 7.3%** | 12.6%** | 9.0%** | 5.5%** | 1.0 (1.9)** |
| Female  (n = 4187) | 40.0% | 15.9% | 20.7% | 21.3% | 21.0% | 20.5% | 17.0% | 18.7% | 13.4% | 12.8% | 20.0% | 11.5% | 21.3% | 1.9 (2.7) |
| Not Reported  (n = 277) | 39.0% | 19.1% | 17.0% | 22.7% | 24.2% | 25.6% | 23.1% | 19.5% | 15.9% | 12.6% | 24.2% | 14.4% | 20.0% | 2.2 (3.0) |
| Age |  |  |  |  |  |  |  |  |  |  |  |  |  |  |
| 11-13 years  (n = 3623) | 27.4%** | 11.7%** | 12.6%* | 14.8%** | 12.3%** | 12.4%** | 11.8%* | 11.7%* | 9.1%** | 8.8%* | 15.0%* | 9.7% | 9.6%** | 1.3 (2.2)** |
| 14-17 years  (n = 5132) | 33.2% | 14.1% | 14.9% | 18.4% | 16.0% | 17.8% | 14.0% | 14.0% | 11.6% | 11.0% | 17.5% | 10.8% | 16.3% | 1.6 (2.5) |
| Nationality |  |  |  |  |  |  |  |  |  |  |  |  |  |  |
| Italian  (n = 7535) | 29.7%** | 12.8%* | 13.7% | 16.1%** | 13.7%** | 15.2%* | 12.4%** | 12.6%** | 10.3% | 9.4%** | 16.0%* | 9.7%** | 13.1%* | 1.4 (2.4)** |
| Other nationality  (n = 1220) | 37.5% | 15.0% | 15.7% | 21.9% | 19.0% | 18.0% | 17.0% | 15.9% | 12.0% | 14.3% | 19.4% | 14.4% | 15.7% | 1.8 (2.7) |
| SHDI |  |  |  |  |  |  |  |  |  |  |  |  |  |  |
| ≤ 85th percentile  (n = 2698) | 33.8%** | 14.9%* | 15.7%* | 19.3%** | 14.5% | 18.2%** | 14.5%* | 15.1%** | 12.3%* | 10.5% | 17.9%* | 12.4%** | 14.2% | 1.7 (2.5)** |
| 90th percentile  (n = 3958) | 30.3% | 12.3% | 12.8% | 16.0% | 15.0% | 14.6% | 12.3% | 12.7% | 10.0% | 10.4% | 16.3% | 9.4% | 13.5% | 1.4 (2.4) |
| 95th percentile  (n = 2099) | 27.9% | 12.4% | 13.9% | 15.4% | 13.5% | 14.0% | 12.7% | 10.9% | 9.5% | 9.0% | 14.9% | 9.5% | 12.5% | 1.4 (2.4) |
| ***Family domain*** |  |  |  |  |  |  |  |  |  |  |  |  |  |  |
| Talking to parents |  |  |  |  |  |  |  |  |  |  |  |  |  |  |
| Ease  (n = 4648) | 22.3%** | 9.8%** | 9.7%** | 13.0%** | 9.2%** | 10.6%** | 8.6%** | 8.9%** | 7.6%** | 7.4%** | 11.8%** | 7.6%** | 7.7%** | 1.0 (2.0)** |
| Difficulty  (n = 4107) | 40.4% | 16.9% | 18.8% | 21.4% | 20.5% | 21.2% | 18.1% | 17.7% | 14.0% | 13.1% | 21.8% | 13.5% | 20.0% | 2.0 (2.7) |
| ***Personality domain*** |  |  |  |  |  |  |  |  |  |  |  |  |  |  |
| Trait Impulsiveness |  |  |  |  |  |  |  |  |  |  |  |  |  |  |
| Above median  (n = 3469) | 43.5%** | 19.7%** | 19.9%** | 24.1%** | 20.7%** | 23.4%** | 19.7%** | 19.1%** | 16.0%** | 15.7%** | 23.1%** | 16.2%** | 19.4%** | 2.2 (2.8)** |
| Below median  (n = 4139) | 18.4% | 6.8% | 8.2% | 9.9% | 8.4% | 8.1% | 6.7% | 7.1% | 5.3% | 4.7% | 10.1% | 4.7% | 7.8% | 0.8 (1.7) |
| Social anxiety disorder |  |  |  |  |  |  |  |  |  |  |  |  |  |  |
| No  (n = 3329) | 13.0%** | 7.2%** | 5.3%** | 8.5%** | 3.4%** | 5.5%** | 4.3%** | 3.7%** | 3.5%** | 3.5%** | 7.0%** | 4.3%** | 3.3%** | 0.6 (1.3)** |
| Yes  (n = 5426) | 41.7% | 16.7% | 19.2% | 22.0% | 21.4% | 21.8% | 18.5% | 18.7% | 14.9% | 14.2% | 22.3% | 14.1% | 19.7% | 2.0 (2.7) |
| Depression Disorder |  |  |  |  |  |  |  |  |  |  |  |  |  |  |
| No  (n = 3655) | 11.1%** | 5.6%** | 4.4%** | 7.2%** | 2.7%** | 4.6%** | 3.7%** | 3.7%** | 3.3%** | 3.1%** | 6.8%** | 4.0%** | 2.2%** | 0.5 (1.2)** |
| Yes  (n = 5100) | 44.9% | 18.5% | 20.8% | 23.8% | 22.9% | 23.6% | 19.8% | 19.7% | 15.8% | 15.1% | 23.4% | 14.9% | 21.5% | 2.2 (2.8) |
| Social withdrawal |  |  |  |  |  |  |  |  |  |  |  |  |  |  |
| Not at risk  (n = 8,582) | 29.9%** | 12.7%** | 13.4%** | 16.2%** | 13.8%** | 15.0%** | 12.4%** | 12.6%** | 10.1%** | 9.7%** | 16.0%** | 9.9%** | 12.7%** | 1.4 (2.4)** |
| At risk  (n = 173) | 76.9% | 32.9% | 42.8% | 52.0% | 49.7% | 46.8% | 44.5% | 35.3% | 33.5% | 36.4% | 41.0% | 33.5% | 54.9% | 4.5 (3.5) |
| Last year academic performance |  |  |  |  |  |  |  |  |  |  |  |  |  |  |
| Failed or lower than the class average  (n = 965) | 41.6%** | 19.5%** | 18.1%** | 25.6%** | 21.1%** | 21.2%** | 19.2%** | 18.9%** | 15.1%** | 17.7%** | 22.9%** | 17.0%** | 19.2%** | 2.2 (2.9)** |
| On average or higher than the class average  (n = 7191) | 28.8% | 12.1% | 13.2% | 15.6% | 13.6% | 14.5% | 12.0% | 12.1% | 9.8% | 9.0% | 15.6% | 9.2% | 12.8% | 1.4 (2.3) |
| Not remembered  (n = 599) | 36.7% | 15.0% | 15.9% | 18.5% | 14.9% | 19.0% | 16.5% | 14.5% | 11.9% | 11.7% | 16.7% | 13.9% | 12.0% | 1.7 (2.5) |
| ***Behavioral domain*** |  |  |  |  |  |  |  |  |  |  |  |  |  |  |
| Social Media Addiction |  |  |  |  |  |  |  |  |  |  |  |  |  |  |
| No  (n = 8515) | 29.5%** | 12.2%** | 13.0%** | 15.8%** | 13.7%** | 14.6%** | 12.2%** | 12.1%** | 9.8%** | 9.4%** | 15.9%** | 9.7%** | 12.5%** | 1.4 (2.3)** |
| Yes  (n = 240) | 74.6% | 45.0% | 46.7% | 54.6% | 41.7% | 50.0% | 45.0% | 46.3% | 36.7% | 34.2% | 37.5% | 32.1% | 47.5% | 4.7 (3.7) |
| Internet Gaming Disorder |  |  |  |  |  |  |  |  |  |  |  |  |  |  |
| No  (n = 7651) | 26.9%** | 11.3%** | 12.5%** | 14.8%** | 12.2%** | 13.3%** | 10.8%** | 11.3%** | 8.6%** | 8.0%** | 13.8%** | 7.9%** | 12.4%** | 1.2 (2.2)** |
| Yes  (n = 1104) | 57.9% | 25.3% | 24.4% | 31.5% | 30.2% | 31.2% | 29.1% | 24.7% | 23.9% | 25.1% | 35.1% | 27.2% | 20.7% | 3.1 (3.1) |
| Doxxing performed |  |  |  |  |  |  |  |  |  |  |  |  |  |  |
| No  (n = 7036) | 28.0%** | 11.2%** | 12.7%** | 14.7%** | 13.0%** | 13.8%** | 11.7%** | 11.8%** | 9.0%** | 8.7%** | 15.1%** | 9.0%** | 12.5%** | 1.3 (2.3)** |
| Yes  (n = 1719) | 42.3% | 20.7% | 19.2% | 25.7% | 20.5% | 23.0% | 18.7% | 17.9% | 16.9% | 15.8% | 22.1% | 15.9% | 17.4% | 2.2 (2.8) |
| Doxxing suffered |  |  |  |  |  |  |  |  |  |  |  |  |  |  |
| No  (n = 6435) | 26.2%** | 11.1%** | 11.8%** | 14.5%** | 11.6%** | 13.0%** | 10.7%** | 10.9%** | 8.7%** | 8.2%** | 13.4%** | 8.4%** | 10.9%** | 1.2 (2.2)** |
| Yes  (n = 2320) | 43.4% | 18.6% | 19.9% | 23.4% | 22.5% | 22.8% | 19.6% | 18.9% | 15.8% | 15.4% | 25.0% | 15.8% | 20.8% | 2.2 (2.9) |
| Online Self-Harm Challenge engagement |  |  |  |  |  |  |  |  |  |  |  |  |  |  |
| No  (n = 8218) | 30.0%** | 12.6%** | 13.5%** | 16.1%** | 13.8%** | 15.1%** | 12.7%** | 12.3%** | 10.1%** | 9.3%** | 15.7%** | 9.6%** | 13.3%* | 1.4 (2.4)** |
| Yes  (n = 537) | 47.3% | 21.4% | 21.6% | 28.5% | 24.4% | 23.6% | 19.4% | 23.5% | 18.4% | 22.7% | 28.5% | 22.3% | 16.8% | 2.5 (3.0) |
| Fruit and Vegetable Diet Habits |  |  |  |  |  |  |  |  |  |  |  |  |  |  |
| Never  (n = 443) | 43.1%** | 22.1%** | 17.4%** | 25.7%** | 20.8%* | 22.8%** | 17.6%** | 20.3%** | 18.3%** | 17.6%** | 21.2%* | 18.5%** | 17.2% | 2.4 (3.2)** |
| Not every week  (n = 1009) | 37.7% | 17.9% | 17.2% | 22.5% | 15.4% | 22.2% | 16.5% | 16.2% | 13.6% | 12.9% | 18.5% | 14.6% | 14.4% | 2.0 (3.0) |
| Weekly  (n = 7303) | 29.1% | 11.9% | 13.3% | 15.6% | 14.0% | 14.2% | 12.3% | 12.1% | 15.9% | 9.3% | 9.3% | 9.7% | 13.1% | 1.5 (2.5) |
| Play competitive sport |  |  |  |  |  |  |  |  |  |  |  |  |  |  |
| No  (n = 5202) | 34.9%** | 14.4%** | 16.1%** | 18.8%** | 16.9%** | 18.7%** | 15.1%** | 14.9%** | 12.0%** | 11.0%* | 18.3%** | 11.4%** | 16.3%** | 1.7 (2.6)* |
| Yes  (n = 3553) | 24.8% | 11.2% | 10.9% | 14.1% | 10.9% | 11.0% | 10.2% | 10.3% | 8.4% | 8.8% | 13.7% | 8.8% | 9.3% | 1.2 (2.1) |
| Volunteering |  |  |  |  |  |  |  |  |  |  |  |  |  |  |
| No  (n = 7205) | 31.4%* | 13.2% | 14.3% | 17.6%** | 14.7% | 15.8% | 13.3% | 13.5%* | 11.2%** | 10.2% | 16.5% | 10.5% | 13.8% | 1.5 (2.4)* |
| Yes  (n = 1550) | 28.0% | 12.6% | 12.5% | 13.5% | 13.4% | 14.5% | 11.9% | 10.6% | 7.6% | 9.9% | 16.3% | 9.7% | 12.1% | 1.3 (2.3) |
| Sleep duration |  |  |  |  |  |  |  |  |  |  |  |  |  |  |
| 6 hours or less  (n = 2671) | 41.5%** | 18.1%** | 19.7%** | 23.6%** | 21.7%** | 22.5%** | 18.3%** | 18.5%** | 21.8%** | 14.3%** | 13.7%** | 14.5%** | 20.9%** | 2.3 (3.1)** |
| 7-8 hours  (n = 4739) | 26.5% | 10.7% | 11.7% | 13.8% | 11.6% | 13.0% | 11.0% | 10.9% | 14.1% | 8.1% | 8.6% | 8.8% | 11.1% | 1.3 (2.3) |
| 9-10 hours  (n = 1024) | 22.9% | 10.8% | 10.3% | 13.6% | 9.1% | 10.3% | 9.1% | 8.8% | 13.3% | 8.1% | 7.9% | 8.6% | 7.4% | 1.2 (2.3) |
| More than 10 hours  (n = 321) | 30.2% | 14.6% | 11.8% | 17.8% | 14.3% | 12.8% | 13.4% | 12.1% | 16.8% | 10.9% | 15.3% | 10.3% | 7.2% | 1.6 (2.6) |
| Sleep latency |  |  |  |  |  |  |  |  |  |  |  |  |  |  |
| Less than 15 minutes  (n = 3904) | 24.4%** | 10.2%** | 10.5%** | 13.6%** | 9.5%** | 12.1%** | 9.8%** | 9.8%** | 13.0%** | 7.4%** | 8.4%** | 8.8%** | 9.3%** | 1.2 (2.3)** |
| 15-45 minutes  (n = 3841) | 33.8% | 14.5% | 15.7% | 18.1% | 16.6% | 17.4% | 14.4% | 11.3% | 17.8% | 11.2% | 11.0% | 11.3% | 14.5% | 1.8 (2.7) |
| More than 45 minutes  (n = 1010) | 43.9% | 18.8% | 20.7% | 25.3% | 25.6% | 22.5% | 20.9% | 21.0% | 25.0% | 16.6% | 15.4% | 14.5% | 25.6% | 2.5 (3.3) |

^a^ Pearson Chi square test. ^b^ Covariates with 2 modalities (Mann–Whitney U non-parametric test), covariates with 3 or more modalities (Kruskal-Wallis non-parametric test). SHDI= Subnational Human Development Index; * p < 0.05; **p <0.01.
